# MicroRNA-194 inhibits epithelial to mesenchymal transition of endometrial cancer cells by targeting oncogene BMI-1

**DOI:** 10.1186/1476-4598-10-99

**Published:** 2011-08-18

**Authors:** Peixin Dong, Masanori Kaneuchi, Hidemichi Watari, Junichi Hamada, Satoko Sudo, Jingfang Ju, Noriaki Sakuragi

**Affiliations:** 1 Department of Gynecology, Hokkaido University Graduate School of Medicine and School of Medicine, Hokkaido University, Sapporo, Japan; 2 Division of Cancer-Related Genes, Institute for Genetic Medicine, Hokkaido University, Sapporo, Japan; 3 Department of Pathology, Stony Brook University Medical Center, NY, USA

## Abstract

**Background:**

Epithelial-mesenchymal transition (EMT) is the key process driving cancer metastasis. Oncogene/self renewal factor BMI-1 has been shown to induce EMT in cancer cells. Recent studies have implied that noncoding microRNAs (miRNAs) act as crucial modulators for EMT. The aims of this study was to determine the roles of BMI-1 in inducing EMT of endometrial cancer (EC) cells and the possible role of miRNA in controlling BMI-1 expression.

**Methods and results:**

We evaluated the expression of BMI-1 gene in a panel of EC cell lines, and detected a strong association with invasive capability. Stable silencing of BMI-1 in invasive mesenchymal-type EC cells up-regulated the epithelial marker E-cadherin, down-regulated mesenchymal marker Vimentin, and significantly reduced cell invasion *in vitro*. Furthermore, we discovered that the expression of BMI-1 was suppressed by miR-194 via direct binding to the BMI-1 3'-untranslated region 3'-UTR). Ectopic expression of miR-194 in EC cells induced a mesenchymal to epithelial transition (MET) by restoring E-cadherin, reducing Vimentin expression, and inhibiting cell invasion *in vitro*. Moreover, BMI-1 knockdown inhibited *in vitro *EC cell proliferation and clone growth, correlated with either increased p16 expression or decreased expression of stem cell and chemoresistance markers (SOX-2, KLF4 and MRP-1).

**Conclusion:**

These findings demonstrate the novel mechanism for BMI-1 in contributing to EC cell invasion and that repression of BMI-1 by miR-194 could have a therapeutic potential to suppress EC metastasis.

## Introduction

Endometrial cancer (EC) is the most frequent gynecologic malignancy in the developed countries [1]. Although the incidence of EC is lower in East Asian than in Western countries, it tends to increase markedly in recent years [2]. EC is generally classified as type I endometrioid EC or type II non endometrioid EC based on etiology and clinical variables. The majority of EC are of type I, which are associated with good prognosis. However, myometrial invasion and distant metastasis decreases the survival rates of patients after surgical treatment. In contrast, type II EC is often related to poor prognostic factors, such as high grade or deep myometrial penetration. Thus, to further improve patient survival, it is essential to further understand the molecular and cellular mechanism of EC development, and in turn, to develop novel therapeutic strategies to block EC progression.

The epithelial to mesenchymal transition (EMT) is a key process contributing to cancer metastasis, characterized by the loss of the epithelial marker E-cadherin, an increase in the mesenchymal markers Vimentin and N-cadherin, and an increase in the migratory and invasive behavior [3]. BMI-1 (B lymphoma mouse Moloney leukemia virus insertion region 1) is a self-renewal gene and overexpressed in multiple human cancers, including lung cancer [4], breast cancer [5], prostate cancer [6], ovarian cancer [7], and recently EC [8]. BMI-1 upregulation is associated with malignant transformation in hepatocellular carcinoma [9]. Notably, recent research has shown that BMI-1 plays essential roles in inducing EMT in head and neck squamous cell carcinoma [10]. However, the roles of BMI-1 in EC metastasis and the molecular mechanism regulating BMI-1 expression remain to be investigated.

Epigenetic alterations (methylation, non-coding microRNA) are critical to cancer development [11]. MicroRNAs (miRNAs) are regulatory, single-stranded non- coding RNAs that repress protein expression by base-pairing with the 3' untranslated region (UTR) of the target mRNA, which triggers either mRNA translation repression or RNA degradation [12]. Aberrant levels of miRNA have been reported in a variety of human cancers, including EC [13]. These observations promote us to hypothesize that certain miRNA may control BMI-1 expression in EC cells, and thus have a therapeutic potential against EC cancer progression.

In this study, we provide experimental evidence that BMI-1 is essential for EMT and invasive phenotype in EC cells. We discovered a novel post-transcriptional regulatory mechanism of BMI-1 expression mediated by miR-194 by directly interacting with the BMI-1 mRNA at the 3'-UTR. The expression of BMI-1 protein level was suppressed by miR-194 with MET transition associated with reduced EC tumor invasion. As a result, it provides a potential new strategy to prevent EC progression by targeting oncogene BMI-1.

## Materials and methods

### Cell lines

Human EC cell lines HHUA (well differentiated), HOUA-I (moderately differentiated) and HEC-50B (poorly differentiated) were obtained from RIKEN cell bank (Tsukuba, Japan) and grown in Minimum Essential Medium Eagle (Sigma-Aldrich, UK) supplemented with 15% of fetal bovine serum (Cambrex Bioscience, Belgium). The cells were maintained at 37°C in a humidified atmosphere of 5% CO_2_.

### Selection of invasive EC cells in transwell invasion chamber

Subpopulations from HEC-50B cells were selected as described previously [14], 6 using transwell plates. Briefly, the polycarbonate membranes (containing 8-μm pores) of the transwell inserts were coated with Matrigel gel (BD Biosciences, CA). Cells were resuspended in serum-free MEM and seeded into the upper wells. MEM medium supplemented with 15% bovine serum was placed into the lower chamber. Following incubation for 24 h at 37°C, the inserts were removed. The cells that migrated through the membranes and attached to the lower-chamber compartments were harvested aseptically and named as HEC-50B-highly invasive (HI)

### Western blot analysis

Whole cellular protein was obtained with M-Per Mammalian Protein Extraction Reagent (Pierce Biotechnology, MA). The aliquots were separated on SDS-PAGE (10%) and transferred to nitrocellulose membranes. Antigen-antibody complexes were detected ECL blotting analysis system (Amersham Pharmacia Biotech, UK). The BMI-1 antibody (ab38295) was purchased from Abcam (Cambridge, MA). E-cadherin antibody (A01589) and Vimentin antibody (A01189) were obtained from GenScript (Edison, NJ). Antibody for p16 (sc-468) and GAPDH (sc-47724) were purchased from Santa Cruz Biotechnology (Santa Cruz, CA).

### Enforced miR-194 expression

HEC-50B-HI cells were transfected with 50 nM of precursor miR-194, or negative control precursor miRNA (Ambion, Austin, TX) by use of Lipofectamine 2000 (Invitrogen, Carlsbad, CA). After 48 h, the cells were processed for Western blot, invasion assay, proliferation, or colony formation assay.

### In vitro cell invasion assay

HEC-50B-HI transfected with precursor miR-194, or negative control precursor miRNA were harvested 48 h after transfection and re-suspended in serum-free MEM. 2 × 10^4 ^cells in 500 μl of MEM medium were added into the upper chamber. In the lower chamber, 750 μl of MEM medium containing 15% fetal bovine serum and 10 μg/ml of bovine fibronectin (Invitrogen, Germany) were placed. The cells were allowed to migrate through the intermediate membrane for 24 h at 37°C. Membranes were then fixed with 10% neutral-buffered formalin and stained in 10% Giemsa solution. The cells attached to the lower side of the membrane were counted in ten high-powered (200×) fields under a microscope. Assays were done in triplicate for each experiment, and each experiment was repeated three times.

### MiRNA quantitative real-time reverse transcription PCR

Total RNA containing small RNA was extracted from cell lines by TRIzol reagent (Invitrogen, CA) according to the manufacturer's instructions. Quantitative reverse transcription (qRT)-PCR was performed to detect the levels of hsa-miR-194 in three EC cell lines by NCode miRNA qRT-PCR Analysis (Invitrogen, CA) according to the manufacturer's protocol. A qRT-PCR forward primer for hsa-miR-194 (5'-TGTAACAGCAACTCCATGTGG-3') was designed and synthesized by Invitrogen. GAPDH was used for normalization [15]. All real-time PCR assays were conducted as previously described [16].

### Luciferase activity assay

The 3'-UTR vector of BMI-1 containing an intact miR-194 recognition sequence was purchased from OriGene Technologies (Rockville, MD). A pGL3 construct containing BMI-1 3'-UTR with point mutations in seed sequence was constructed using a QuickChange site-directed mutagenesis kit (Stratagene, CA), using the following primers: 5'-CATTACTTTTACATATATTGCTGGCCCTTCTGCTTTC-3' (forward) and 5'-GAAAGCAGAAGGGCCAGCAATATATGTAAAAGTAATG-3' (reverse). Cells were transfected with 50 nM precursor miRNA (miR-194, miR-128 or control miRNA) along with the wild-type or mutant BMI-1 3'-UTR-luciferase constructs. 24 hours after transfection, luciferase activity was measured using dual-luciferase assay (Promega, Madison, WI).

### BMI-1 silencing by shRNA

BMI-1 shRNA plasmids (sc-29814-SH) and control shRNA plasmids (sc-108060) were obtained from Santa Cruz Biotechnology (Santa Cruz, CA). HEC-50B-HI cells were stably transfected as described previously [17]. In brief, cells at 70% confluency were transfected with Lipofectamine PLUS Reagent (Invitrogen, CA) according to the manufacturer's protocols and selected in MEM medium containing puromycin (Sigma-Aldrich, MO) at 1 μg/ml 48 h post-transfection. Selected clones of HEC-50B-HI cells were expanded into HEC-50B-HI-BMI-1 shRNA cells and HEC-50B-HI control shRNA cells, respectively.

### MTT assay

For measurements of cell proliferation rates, 1 × 10^3 ^cells were plated into each well of 96-well plates and cultured in 100 μl of medium containing 10% serum. After 1 or 5 days incubation, 10 μl of MTT solution (Cell counting kit-8, Dojindo, Japan) 10 was added into each well, and plates were incubated for 4 h at 37°C, and 450 nm UV absorbance of each sample was measured in a microplate reader. Assay was done in triplicate wells, and each experiment was repeated three times.

### Colony formation assay

About 500 cells were seeded per well in 6-well plates. After 10 d, the cells were fixed in 70% ethanol and stained with 10% Giemsa (Sigma- Aldrich, MO). Colonies consisting of >50 cells were counted. The results represented the average of three independent experiments.

### qRT-PCR of stemness markers and chemoresistance genes

Real-time RT-PCR was performed using a Primescript One Step RT-PCR Kit (Takara, Japan). Primers used for SOX-2, KLF4, MRP-1 and GAPDH were previously reported [18].

### Statistical analysis

All experiments were performed in triplicates. Statistical analyses were performed using SPSS statistical software. Student's t-test was adopted. Significance was 11 defined as *P *< 0.05.

## Results

### BMI-1 expression correlates with invasive potential and EMT phenotype of EC cells

**Figure 1:**
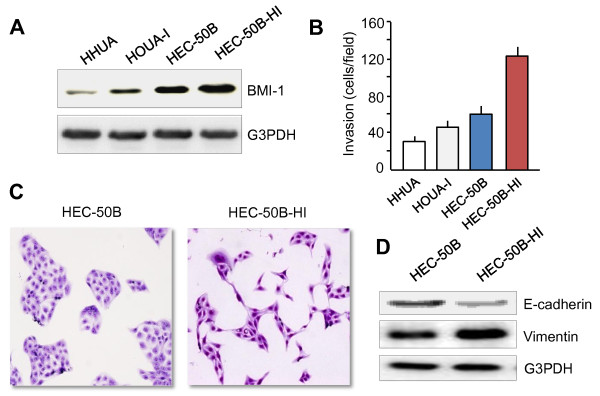
**Association between BMI-1 expression and invasive potential and EMT features of EC cell lines**. **A**. BMI-1 protein levels in the panel of EC cell lines were determined by Western blot analysis. **B**. Invasive ability of EC cells was evaluated by Matrigel invasion assays. Data represent the mean ± SE of three independent experiments. **C**. Highly invasive HEC-50B-HI cells exhibiting fibroblastic morphology and the parental HEC-50B cells showing epithelial-like appearance were fixed with neutral-buffered formalin, stained in Giemsa, and images were taken (magnification × 100). **D**. Western blot analysis of epithelial marker E-cadherin and mesenchymal marker Vimentin in HEC-50B and HEC-50B-HI cells.

BMI-1 is thought to mediate cell invasion in several types of cancer. To assess the role of BMI-1 in conferring invasive properties to EC cells, we used Western blot analysis to characterize expression of BMI-1 in four human EC-derived cell lines (HHUA, HOUA-I, HEC-50B and HEC-50B-HI), and determined whether expression of endogenous BMI-1 correlated with invasive ability by using an *in vitro *cell invasion assay. In contrast to HHUA cells with markedly reduced endogenous BMI-1 expression, HEC-50B-HI cells expressing 5-fold higher levels of BMI-1 protein exhibited the highest invasive potential after 24 h incubation (Figure 1A). The number of HEC-50B-HI cells that passed through the membrane was 5.0 times larger than the number of HHUA cells (Figure 1B), thus suggesting that BMI-1 expression level seemed to be closely associated with the enhanced invasive activities of EC cell lines.

It has been suggested that EMT plays a crucial role in cancer metastasis [3]. 12 During the acquisition of EMT characteristics, cancer cells lose the intercellular junctions and gain fibroblast-like motile and invasive phenotype, associated with the down-regulation of epithelial marker E-cadherin and the up-regulation of mesenchymal marker Vimentin [3]. To test the hypothesis that BMI-1 would be involved in the acquisition of an invasive EMT phenotype in EC cells, we evaluated the morphological changes between HEC-50B-derived aggressive sub-cell lines and its parental HEC-50B cells. Highly invasive HEC-50B-HI cells displayed spindle-like, fibroblastic morphology when comparing to HEC-50B cells showing epithelial-like appearance (Figure 1C). In accordance with this finding, expression of E-cadherin was significantly reduced in HEC-50B-HI cells. In contrast, expression of Vimentin was significantly increased in this cell line (Figure 1D), demonstrating that up-regulation of BMI-1 may contribute to EMT-derived invasive phenotype in EC cells.

### Silencing of BMI-1 expression reverts EMT phenotype and reduces EC cell invasion

**Figure 2:**
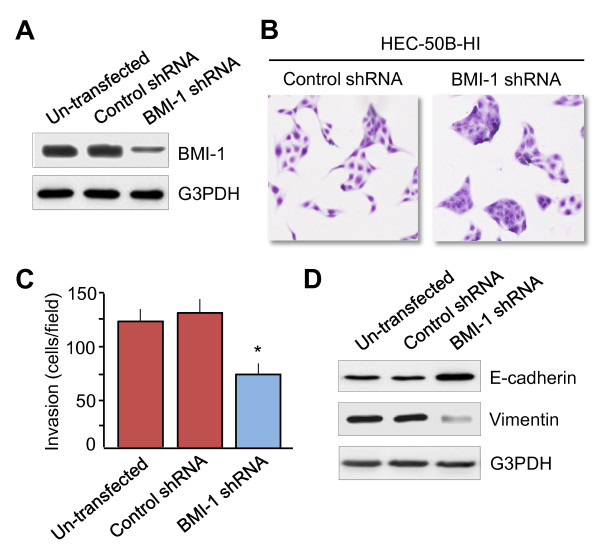
**Silencing of BMI-1 expression reverts EMT phenotype and reduces EC cell invasion**. **A**. Comparison of BMI-1 levels in un-transfected HEC-50B-HI, HEC-50B-HI-BMI-1 shRNA and control shRNA cells. **B**. Morphological changes of HEC-50B-HI cells after knockdown of BMI-1 expression 27 (magnification × 100). **C**. Effects of BMI-1 suppression on cell invasion ability using Matrigel invasion assay. Data represent the mean ± SE of three independent experiments. *P < 0.01. **D**. Western blots of epithelial marker E-cadherin and mesenchymal marker Vimentin in un-transfected HEC-50B-HI, HEC-50B-HI-BMI-1 shRNA and control shRNA cells.

To validate whether BMI-1 affects EMT and cell invasion of EC cells, HEC-50B- BMI-1 shRNA and control shRNA cell lines were established. The BMI-1 protein level was significantly decreased in BMI-1 shRNA-transfected HEC-50B-HI cells (Figure 2A). Knockdown of BMI-1 protein seemed to change the morphology from spread fibroblastoid to epithelial-like appearance (Figure 2B), decreased cell invasion (Figure 2C), and resulted in the up-regulation of E-cadherin and down-regulation of Vimentin at protein levels (Figure 2D). These results suggest that BMI-1 is critical for the acquisition of EMT characteristics in EC cells.

### MiR-194 directly targets BMI-1, and reverses EMT phenotype in EC cells

**Figure 3:**
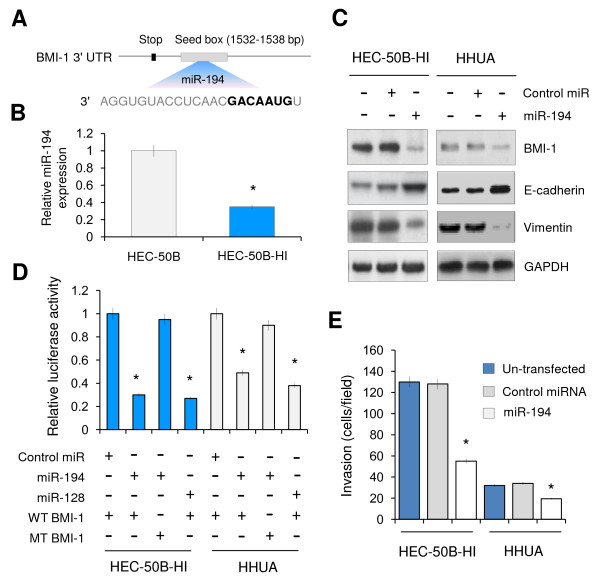
**MiR-194 directly targets BMI-1, and reverses invasive, EMT phenotype in EC cells**. **A**. Sequence of miR-194 binding site in the BMI-1 3'UTR predicted with TargetScan. **B**. MiR-194 expression levels in HEC-50B and HEC-50B-HI cells were determined by Real-time RT-PCR. Data represent the mean ± SE of three independent experiments. *P < 0.01. **C**. Western blotting analysis of BMI-1, E-cadherin and Vimentin in un-transfected EC cells and EC cells transfected with miR-194 or control miRNA. **D**. HEC-50B-HI and HHUA cells were transfected with WT or MT BMI-1 3'UTR luciferase vectors, along with miR-194, miR-128 or control miRNA. Luciferase activity was measured at 24 h after transfection. Data represent the mean ± SE of three independent experiments. *P < 0.01. **E**. Invasive potential of un-transfected and EC cells transfected with miR-194 or control miRNA was evaluated by Matrigel invasion assays. Data represent the mean ± SE of three independent experiments. *P < 0.01.

Based on miR target analysis using TargetScan website [19], miR-194 was identified as potential regulator of BMI-1 expression. The predicted binding of miR-194 with BMI-1 3'UTR was illustrated (Figure 3A), indicating that miR-194 is a potential miRNA targeting BMI-1. To compare the relationship between miR-194 and BMI-1 expression, we examined the endogenous miR-194 expression level by qRT-PCR in HEC-50B-HI and HEC-50B cells. MiR-194 mRNA expression was significantly lower in high BMI-1 expressing HEC-50B-HI cells (Figure 3B), showing that miR-194 level inversely correlated with BMI-1 expression. To examine the inhibitory effect of miR-194 on BMI-1 protein level, we performed Western blot analysis at 48 h after miR-194 transfection into HEC-50B-HI and HHUA cells. Ectopic expression of miR-194 in these two cell lines significantly decreased the protein levels of BMI-1 compared to 14 control miRNA (Figure 3C).

To further confirm that BMI-1 is the direct target of miR-194, firefly luciferase reporter vector containing a segment of the 3'UTR of BMI-1 with point mutations in the seed sequence was constructed. In addition, miR-128 has been reported to target BMI-1 in glioma [20]. Therefore miR-128 was used as a positive control. The BMI-1 luciferase constructs were then co-transfected with miR-194, miR-128 or control miRNA into HEC-50B-HI as well as HHUA cells. As expected, transfection with miR-128 dramatically attenuated BMI-1 3'-UTR luciferase activity. Interestingly, the BMI-1 3'-UTR luciferase activity in miR-194-transfected both HEC-50B-HI and HHUA cells was significantly lower when comparing with control miRNA-transfected cells, whereas mutation of miR-194 recognition site rescued the luciferase activity (Figure 3D). These results collectively suggested a direct and specific inhibition of miR-194 on BMI-1 3'-UTR in EC cells.

BMI-1 has been shown to promoting EMT in head and neck squamous cell carcinoma [10], implies the possibility that reexpression of miR-194 may lead to a reversal of EMT phenotype of invasive EC cells. To test our hypothesis, the effect 15 of ectopic expression of miR-194 on cell invasion and expression of EMT markers was investigated in HEC-50B-HI and HHUA cells. Transfection with miR-194 significantly decreased cell invasion in these cells. However, this inhibition was more evident in HEC-50B-HI cells (Figure 3E). Furthermore, transfection of miR-194 induced a loss of the mesenchymal phenotype by restoring epithelial marker E-cadherin and reducing mesenchymal marker Vimentin expression (Figure 3C). These results indicated that reexpression of miR-194 could reverse the EMT phenotype, and dramatically decreased cell invasion in BMI-1 expressing EC cells.

### Knockdown of BMI-1 repressed *in vitro *cell proliferation and clonal growth

**Figure 4:**
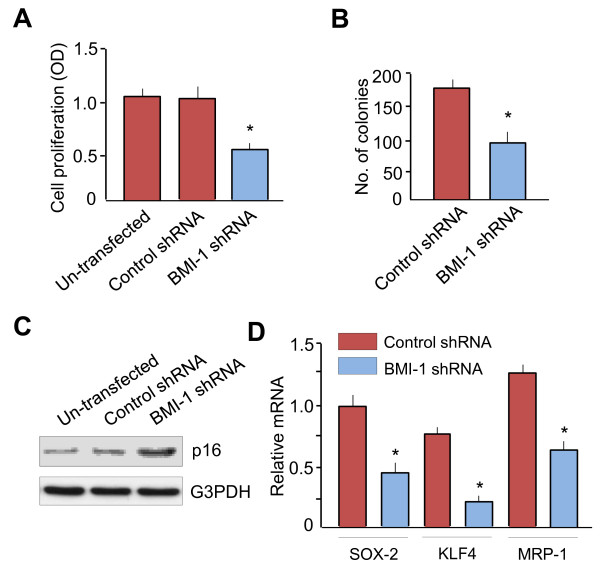
**Knockdown of BMI-1 repressed *in vitro *cell proliferation and clone growth**. **A**. Effects of BMI-1 repression on cell proliferation assessed by MTT assay in un-transfected HEC-50B-HI cells, HEC-50B-HI-BMI-1 shRNA and control shRNA cells. *P < 0.05. **B**. Colony formation was assessed following stable repression of BMI-1. *P < 0.05. **C**. Western immunoblot analysis of p16 protein expression in un-transfected HEC-50B-HI cells, HEC-50B-HI-BMI-1 shRNA cells and control shRNA cells. **D**. mRNA expression levels of SOX-2, KLF4 and MRP-1 in HEC-50B-HI-BMI-1 shRNA and control shRNA cells were determined by Real-time RT-PCR. Data represent the mean ± SE of three independent experiments.

It has been shown that BMI-1 is required for the clonogenic growth of multiple myeloma cells [21]. Therefore, we examined the roles of BMI-1 in regulating proliferation and colony growth of HEC-50B-HI cells. As expected, cell proliferation was significantly decreased in BMI-1 shRNA-transfected cells compared with control cells (Figure 4A). Moreover, colony formation of HEC-50B-HI cells was significantly reduced after BMI-1 protein expression was effectively suppressed by shRNA transfection (Figure 4B). Since previous studies have shown that BMI-1 promotes cell proliferation through suppression of tumor-suppressor p16 [22], we determined the p16 protein level by Western blot. Down-regulation of BMI-1 expression by BMI-1 shRNA transfection significantly increased p16 protein expression (Figure 4C). These results indicated that knockdown expression of BMI-1 could suppress EC cell proliferation and colony growth in EC cells by upregulation of p16 expression.

Overexpression of BMI-1 in head and neck squamous cell carcinoma promote stemness properties by increasing the expression of stem cell marker and drug-resistance gene [18]. By using qRT-PCRs, we demonstrated that the mRNA expression levels of stemness genes SOX-2, KLF4 and chemoresistance gene MRP-1 were downregulated in HEC-50B-HI-BMI-1 shRNA cells compared with control shRNA cells (Figure 4D). These results suggest the important roles of BMI-1 in modulating cell proliferation and stemness in EC.

## Discussion

Previous studies have linked BMI-1 upregulation to enhanced cell invasion in various human tumors [4-7]. However, the role of BMI-1 in EC cell invasion has not been thoroughly explored. In this study, we discovered that elevated BMI-1 expression closely associated with EC cell invasion. In line with our study, up- regulation of BMI-1 has been shown to induce breast cancer metastasis [23]. Similarly, silencing of BMI-1 can inhibit the metastatic capability of cervical cancer cells [24]. Our results provided new evidence to support the oncogenic roles of BMI-1 in enhancing EC metastasis.

The acquisition of EMT-derived phenotype confers invasive behavior of tumor cells. BMI-1 is able to induce EMT in nasopharyngeal epithelial cells by repressing E- cadherin expression and up-regulating Vimentin expression [25]. We showed for the first time that knockdown of BMI-1 in EC cells led to an attenuation of *in vitro *invasion and a reversion of EMT, evidenced by epithelial-like morphology, increased expression of E-cadherin and decreased expression of Vimentin. These findings highlight the pivotal role for BMI-1 in driving invasive potential in EC cells through induction of EMT phenotype.

Dysregulation of miRNAs are implicated in EMT modulation [26]. MiRNAs, such as miR-200 family and miR-205, act as key modulators of EMT and enforcers of the epithelial phenotype [27]. We showed that miR-194 suppress BMI-1 expression through direct interaction and inhibited EC cell invasion. These results were consistent with a recent report that miR-194 reduces invasion of the mesenchymal-18 like liver cancer cells both *in vitro *and *in vivo *[28]. The finding that BMI-1 expression was also reduced by overexpressing miR-194 in HHUA cells with low level of BMI-1 may reflect the heterogeneity of EC. Sub-populations of this cell line might be more aggressive and exhibit higher BMI-1 expression. Thus, targeting BMI-1 in invasive EC cells by miR-194 would be valuable in development of new therapeutic strategies against EC metastasis.

Cancer stem cell (CSC) has been demonstrated to play important roles in cancer metastasis [3]. In many CSC populations, aberrant BMI-1 expression is reported [29]. CSC isolated from EC has been shown to express BMI-1 [30]. We demonstrated that knockdown of BMI-1 in invasive mesenchymal EC cells significantly reduced in cell proliferation, colony growth and up-regulated the p16 tumor suppressor. Many independent studies also support the association between BMI-1-dependent p16 suppression and enhanced stem cell proliferation [22]. Moreover, we demonstrated that loss of BMI-1 in EC cells reduces expression of stemness gene SOX-2, KLF4 and drug-resistance gene MRP-1, suggesting that BMI-1 expression maybe required for the proliferation of cancer cells, as well as for regulation of stemness properties of EC cells.

In conclusion, we discovered a critical function of BMI-1 to potentiate EC metastasis via induction of EMT. We also revealed a new regulatory mechanism of BMI-1 expression by miR-194 to inhibit EC cell invasion through down-regulation of BMI-1. As a result, restoration of miR-194 expression could have important implications for the clinical management of EC.

## Competing interests

The authors declare that they have no competing interests.

## Authors' contributions

DP and JJ designed research; DP carried out the molecular genetic studies; DP, KM, HJ and SN analyzed data; DP and JJ wrote the paper. All authors read and approved the final manuscript.
